# Triphenotypic multiple myeloma expressing kappa or lambda light chain or both

**DOI:** 10.1111/bjh.12100

**Published:** 2012-10-30

**Authors:** Yang Liu, Yan Zhang, Weidong Han

**Affiliations:** 1 Department of Geriatric Haematology, Chinese PLA General HospitalBeijing, China; 2 Bio-therapeutic Department, Chinese PLA General HospitalBeijing, China

**Figure 1:**
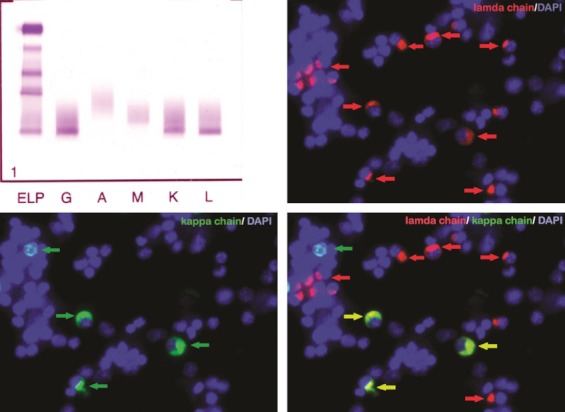


A 70-year-old man presented with a 6-year history of oedema of both lower extremities. Ultrasonic examination showed bilateral anterior tibial artery occlusion due to thrombosis, moderate bilateral pleural effusions and ascites. He had severe pancytopenia, chronic renal dysfunction (serum creatinine 223·8 μmol/l and more than 1 g/24 h of urinary protein) and, on ultrasound examination, mildly enlarged kidneys showing diffuse damage. Serum and urine electrophoresis with immunofixation revealed both monoclonal IgG lambda and monoclonal IgG kappa immunoglobulins (top left). Serum calcium and phosphate were normal, as were the results of a radiographic skeletal survey. Wright's staining of a bone marrow film showed that plasma cells accounted for 13% of nucleated marrow cells. Bone marrow mononuclear cells were enriched by density gradient centrifugation and dual immunofluorescence staining then showed that 13%, 7% and 6% of mononuclear cells were positive for lambda alone (red arrows), kappa alone (green arrows) or both lambda and kappa light chains (yellow arrows), respectively (top right, bottom left and bottom right). Cells staining for lambda alone, kappa alone or both lambda and kappa were also demonstrated in ascitic fluid. A diagnosis of multiple myeloma with triphenotypic characteristics (kappa alone, lambda alone, and both) was made. After initial treatment with vincristine, doxorubicin and dexamethasone (VAD) regimen, the patient' condition stabilized, and after 1 year's follow-up he continued treatment with thalidomide alone.

